# Does public health policy quality foster state innovation capacity? Evidence from a global panel data

**DOI:** 10.3389/fpubh.2022.952842

**Published:** 2022-11-10

**Authors:** Xiaoyi Ji, Ling Gao, Huan Liu, Shengyu He, Baoqing Zhu, Cheng Chow, Jieqiong Chen, Zhipeng Lu, Li Li

**Affiliations:** ^1^Faculty of Innovation and Entrepreneurship, Wenzhou University, Wenzhou, China; ^2^Wang Yanan Institute for Studies in Economics, Xiamen University, Xiamen, China; ^3^School of Business Administration, Zhejiang Gongshang University, Hangzhou, China; ^4^School of Public Affairs, Zhejiang University, Hangzhou, China; ^5^School of Marxism, Fudan University, Shanghai, China; ^6^Department of Social Work and Social Administration, The University of Hong Kong, Pokfulam, Hong Kong SAR, China; ^7^Department of Political Science, Party School of Zhejiang Provincial Committee of C.P.C, Hangzhou, China

**Keywords:** policy quality, state innovation capacity, public health, health service efficiency, post-pandemic era

## Abstract

The design and implementation of public health policy may shape state innovation capacity with governance effectiveness, political stability, and government integrity. Previous studies, however, failed to incorporate these relationships simultaneously. This study aims to combine two distinct scholarships to examine whether the quality of policies in the public health sector contributes to state innovation capacity. We extracted data from the WHO international health regulatory dataset covering the WHO Member States between 2010 and 2017 to investigate the relationship (*N* = 145). Our fixed-effects models and regression discontinuity design (RDD) suggest a positive impact of public health policy quality on state innovation capacity. There are several contributions to the study of the relationship between public health and innovation in this study. Firstly, it fills a theoretical void concerning the relationship between policy development and implementation in the public health sector and country-specific innovations. Second, it provides an empirical quantitative analysis of policy quality in the public health sector. Third, this study contributes evidence that public health plays an important role in fostering state innovation beyond urbanization, investment in science and technology, and foreign trade. Furthermore, our quasi-experimental evidence found that this mechanism may be significant only between the more politically stable countries and the most politically stable countries. These contributions have empirical implications for governments across the world that seek to balance public health and innovation capacity in the context of the post-pandemic era.

## Introduction

Innovation is generally defined as “a new idea, knowledge, technology, product, policy, process, or practice that an individual or organization of adoption considers being new” (1). Technological investment, urbanization, organizational culture and foreign trade are crucial to innovation (2, 3), especially in an uncertain social environment, which is considered a favorable opportunity for innovation (4). The outbreak of the COVID-19 epidemic has brought countries around the world into an uncertain economic and social environment, and the global and rapidly spreading virus has placed “unprecedented demands on the health systems of most countries in the world” (5). The global pandemic also highlights how governments design and implement different policies to mobilize medical resources (6). As government spending on health care has increased, the question of improving state innovation capacities has gradually been raised in the post-pandemic era. When compared with factors such as research and development (R&D), talent, taxation, and trade that drive state innovation, the public health and healthcare factors in the era of the pandemic render this question more puzzling than ever. Whether it is a country with a policy of coexistence with COVID-19 or a country with a policy of “dynamic zero-COVID” to eliminate the risk of transmission, they are faced with 5–10 times more costs for building a public health system than what they have in the past (7). Public health policies implemented by countries throughout the world, however, may promote socio-economic development with positive spillover effects.

The outbreak of public health crises has increased the level of government support and attention to public health policies (8–11). It is becoming increasingly important to improve and diversify healthcare delivery, which has attracted the attention of academia and international organizations that place an emphasis on this issue. As noted by organizations such as WHO, a strong public health system is a prerequisite of effective social governance in times of public health crisis (12), and a strong public health system requires support from strong institutions and sound policy (13). Globally, different health systems are implementing different approaches to promote policy quality improvement (14, 15). An evaluation of the policy quality improvement strategies of the 25 member states of the European Union (EU) revealed that many of these countries have implemented performance indicators, Total Quality Management (TQM), and systems for obtaining public comments. Public health policies can be improved by utilizing these strategies, and they appear to be most effective when used in tandem (16–18). However, there are many low-income countries that have failed to adopt effective strategies to improve the quality of policies in the public health sector. We therefore aim to uncover the relationship between the quality of public health policy and state innovation capacity in order to recommend that countries around the world pay attention to the formulation and implementation of public health policies. We suggest that policy quality in public health is a relevant factor in governance and can contribute substantially to state innovation capacity, and this intuition is supported by an analysis of a subset of the literature on the relationship between policy and innovation (19–25).

The public policies that influence state innovation include distinct policies and policy toolkits launched at different times and with diverse motivations along with a dynamic dialogue with government effectiveness (26, 27). Contemporary mainstream economists and policy scholars believe that public policy affects state innovation capacity through two approaches. A primary aspect of the policy is the mission orientation, which seeks to provide innovative solutions to challenges on the political agenda as well as make a difference in practice. To ensure that the proposed solutions are feasible in practice, policymakers ought to take into account all stages of the innovation process in developing and implementing policies. When it comes to national defense, while some government policies do not directly drive innovation in other areas, many innovations (e.g., internet development) with significant socio-economic implications are a consequence of the effective governance of national defense policies (28–30). Similarly, public policies to address the public health crisis may also promote innovation in other spheres, under the influence of the public health crisis (31, 32). A second policy effect is the diffusion of innovative derivatives, which can be invented or developed through government policy research and development. As policymakers around the world began believing that advances in science and technology could potentially benefit society in the early post-World War II era, the inventive orientation of policy was widely adopted (33–36). These policies are commonly referred to as innovation policies, and they are particularly prevalent in the sphere of public health and health care (37–39), in which public health policy executors utilize innovation to bring potential benefits to society (public goods), such as improving social stability or intangible infrastructure (40–43). Therefore, our research question is: Does the improvement of the quality of public health policies have an impact on the enhancement of state innovation capacity?

Considering both of these approaches together, it is prudent to assume a positive relationship between the quality of public health policy and the state innovation capacity. Despite this, there are few studies describing this special relationship; that is to say, on the one hand, we did not find any theoretical or empirical evidence to investigate the relationship between public health policy quality and state innovation capacity, and on the other hand, we did not find studies that discussed moderators, such as government effectiveness and social stability (44). Toward advancing research on the impact of public health policy and state innovation capacity, we constructed an empirical model that used state innovation capacity as the outcome variable and public health policy quality as the explanatory variable. To estimate our model, we selected three moderating variables according to the existing literature on the relationship between public policy and innovation: (1) Government effectiveness: An improvement in public health policy enhances government effectiveness, and this, in turn, enhances innovation capacity within states (45, 46); (2) Political stability: improvements in public health policy shape a stable political environment in the country, and political stability enhances national innovation capacity (47, 48); (3) Government integrity: increasing investment in areas of public health policy with a narrow rent-seeking space reduces the possibility of rent-seeking at the state level while enhancing state innovation capacity increases integrity at the state level (49–52). To strengthen the estimation of the relationship between independent and dependent variables, we also examined factors of interest in the conventional policy literature, such as economic growth rates, urbanization, and trade.

Our empirical analysis is based on data from WHO International Health Regulations (IHR) for all WHO Member States (to attain a balanced panel, we selected 145 of them), and covers the period 2010 through 2017. The main results panel regression indicates a positive relationship between public health policies and state innovation capacity, and the government can raise state innovation capacity by improving the quality of public health systems and policies. We did not find any evidence to suggest that government policies have any significant influence on public policy quality, however, both political stability and government integrity can bolster their relationship.

The structure of this study is as follows. The next section reviews available literature on policy quality and state innovation and proposes our research hypotheses. The paper then describes the data and method in section 3. Next, we present the main findings of our study and discuss the main results to highlight the impacts of public health policy quality in section 4. Finally, we summarize the policy implications and provide directions for future research.

## Literature review

### Public health policy quality and state innovation capacity

A number of scholars in the field of health economics, health governance, and public health policy have produced extensive work on the quality of public health policy since the welfare state became a manifestation of contemporary high-income countries (21, 53, 54), and it remains a prevalent topic in the fields of health economics, health governance, and public health policy (55, 56). Public health policy quality was blended with medical innovation, medical entrepreneurship, and medical diplomacy to explore the impact of these factors on improving health, economic growth, and sustainable development (57, 58), as well as the positive relationship between innovations policies for the public health sector and health governance objectives (59).

Despite the significant amount of literature that exists on public health policy quality and innovation policy, respectively, and that literature is increasing rapidly in the context of global public health crises (57), there have been few studies that combine the two seemingly distinct areas of research. There may be two important reasons for this: (1) Prior to the public health crisis, government expenditures in the field of public health accounted for a small share, so the government and academia did not recognize its link to innovation; (2) There are no objective indicators to measure the effectiveness of public health policies.

Our theoretical argument is illustrated in this study by exploring the mediators between public health policy and state innovation capacity in accordance with Bloom, Van Reenen, and Williams (60), who provide an overview of how state innovation may be promoted using a combination of different policy instruments. Likewise, we consider that all policies are, fundamentally, policy toolkits consisting of sub-policies in different spheres of governance and that public health policy encompasses not only the field of public health but the integration of policy tools in many spheres of governance. As part of the process of establishing and implementing public health policies, state innovation capacity is impacted by several mechanisms.

Firstly, effective public health policies strengthen long-term innovation strategies and allow for more accurate investment and implementation of measures to ensure their success. Bonander and Gates (61) pointed out that public health policies offering more informationalized services could provide opportunities for the development of health monitoring and outbreak management, which are considered state capacity. And most scholars came to the conclusion that an improved public sanitarian regime can benefit the construction of web-based infrastructure in the field of public-private health management and point-to-point investment (62, 63). Meanwhile, as a typical public good, public health policies seem to have positive externalities to some extent, because of their spillover effect on other fields systematically. Mushkin (64) and Grossman (65) have argued that a higher public health expenditure can bring a healthier human capital, and eventually can result in a higher-efficiency economy where the mechanism is that the individual invests in human capital will promote economic productivity including innovation.

Moreover, the innovation preference could be influenced by the forms of public health policies. For example, facing the challenge of transmitting some diseases like HIV, governments, when during the policy-making period, would be more likely to take a long-term innovation strategy to prevent it (66). Secondly, in an environment of social stress characterized by public health crises, the investment propensity of the private sector is influenced by health policy regimes. Effective public health policies contribute to the emergence of novel inventions, new technologies, and new products. Furthermore, when the government's investment in public health has increased significantly, effective public health policies reduce the risk of rent-seeking and improve innovation outcomes. In addition, there is a positive relationship between relationship governance and innovation performance (67). Accordingly, it is hypothesized that the government, at the state level, can improve state innovation capacity by enhancing its public health policy.

***Hypothesis 1: State innovation capacity is positively influenced by the quality of public health policies*.**

This study intends to analyze state-level panel data, and therefore it is necessary to determine how to measure, track, or quantify the quality of public health policy. Despite the presence of at least some data on these elements, the problem is often found in the databases that store them because they are often incomplete, with very limited access to country-level data. Consequently, it is generally believed that there are no flexible tools to measure the outcomes of health care (68). We address this question by describing the content of each public health system and policy and then comparing the content to the WHO database, the only global database that contains public health data. With regard to public health institutions, we apply the perspective of the neo-institutionalist, accounting for both formal and informal institutions, norms, and relationships within the public health sector (69–71). Therefore, the public health system we study is not merely a formal organization, but also includes customs and traditions related to public health within a country. A public health plan or action plan is a strategy or procedure for guiding the actions of public health personnel to provide health care services to society (72). To understand the effect of public health policy, we turn to the rational choice theory's definition of policy, which refers to the government's approach to solving public health issues. It is generally believed that public health policies can help improve the effectiveness of social governance and improve the degree of social stability, which corresponds to the hypothesis of the moderator variables in this study (73). The institutions and policies of public health are therefore situated within the invisible dimensions of a state's public health care system.

The second issue relates to the measurement of state innovation capacity. Since the introduction of innovation measurement, it has become widely accepted by the public economics community. The role of various types of policies in encouraging and protecting state innovation has been studied extensively by academics and policymakers (2, 74–76). Currently, the current academic environment is in disagreement regarding innovation measurement, but one standard that has been uncontroversial is the number of patents. The state innovation input and innovation output are closely tied to the state innovation capacity, and patents are regarded as a measure of state innovation capacity (77). The most commonly used innovation input indicators are the political environment (78), human capital and R&D resources (79), market maturity (80), and business maturity (81). Additionally, innovation output indicators commonly include knowledge and technology output (for example, the number of patents) (82–84) and creative output (85).

This study holds the position that state innovation capacity consists of state innovation inputs and state innovation outputs, in order to include all aspects of state innovation capacity and to control for endogeneity (86).

### Moderation effects of government effectiveness, political stability, and government integrity

As described above, much of the previous literature has found a significant relationship between quality public health policy and governance effectiveness, political stability, and government integrity, or between these factors and quality public health policy (45–52). However, these moderators have not been included in research concerning the relationship between public health policy and state innovation capacity.

The purpose of public policy is to ensure the effective governance of society by governments. The improvement of public policies in the field of public health is therefore a realization of government effectiveness. Public health policies that promote government efficiency and effectiveness also promote state innovation capacity, creating a mutually beneficial relationship (45); Effective governance involves three factors, including the efficient use of public-private partnerships (PPPs) or community networks of organizations and cooperation; the effective application of market mechanisms to allocate resources in accordance with the principles of market competition, while operating under government supervision; effective management of government and state bureaucracies from the top-down. The PPPs, market, and government have guaranteed the input and output of science and technology at the institutional and policy levels, as well as enhanced state innovation capacity (46). In light of this, we propose the following hypothesis:

***Hypothesis 2: Public health policies have a greater positive impact on state innovation capacity in countries with higher governance effectiveness*.**

Institutional economists generally hold that good public policy provides the public with effective public goods, which enhances the satisfaction of the public with the government and increases its legitimacy for the government. Public health policies, specifically epidemic control and public health systems, have a substantial impact on the satisfaction of the public in times of public health crises (47). In this regard, the quality of public policies relating to public health is also a significant factor in establishing a favorable political environment for a state, namely, a stable regime climate. The development of science and technology within a country and the exchange of science and technology between nations require a stable political climate (48). Accordingly, the following hypothesis is formulated:

***Hypothesis 3: Public health policy quality has a greater positive effect on state innovation capacity in countries with higher political stability*.**

Numerous studies have shown a negative correlation between government rent-seeking and the quality of public policy. The phenomenon of government rent-seeking has been effectively curbed when public policy formulation and implementation supervision have been strengthened (50, 51). With rent-seeking costs on the rise, manufacturers are shifting their capital investments from rent-seeking to innovation. Therefore, reducing the space available for government rent-seeking improves the state's capacity for innovation (49, 52). Based on this, the following hypothesis is made:

***Hypothesis 4: The quality of public health policies has a more pronounced positive impact on the level of the state innovation capacity of states with a higher level of government integrity*.**

However, the boundaries between corruption and incorruptibility, stability and instability, efficiency and inefficiency are not clear. Therefore, it is difficult to directly claim that the implementation of high-quality public health policies in clean (stable or efficient) countries will inevitably lead to innovation. Korea, which was once corrupt and backward, also had miracles as a developmental state (87). And countries caught in the European debt crisis also have the problem of low investment efficiency caused by rent-seeking (88). Therefore, here we put forward the next assumption:

***Hypothesis 5: we assume that the more stable countries and the most stable countries have more significant differences in the promotion of GII by policy quality*.**

## Data and method

### Data description

There are a number of indicators that indicate the level of innovation at the national level, such as the Regional Innovation Index (OECD), the Global Innovation Index (GII), the Global Entrepreneurship Index (GEI), and the Regional Entrepreneurship Development Index (REDI). Additionally to the standard criticism of these indicators, another problem arises from the fact that they often fail to capture all the important characteristics resulting from the simple collection of data, and therefore do not provide a comprehensive picture of state innovation (89). A second caveat is that some of these indicators (REDI, GEI) are inapplicable as secondary and tertiary indicators in these indices overlap with independent variables and moderator variables, creating endogeneity. While some exceptions attempt to address endogenous issues (such as the OECD-RII), they neglect the two-dimensionality of state innovation. Consequently, we choose the GII index which avoids endogeneity and presents two dimensions of the national innovation capability as a database. Referring to other quantitative studies on public health policy, we used the International Health Regulations (IHR) Core Competency Index as a basis for measuring the Quality of Public Health Policy.

The sample we examined covered almost the entire population of each of the 192 members of the World Health Organization throughout an 8-year period from 2010 to 2017. Since data were unavailable in some countries for some years, we selected 145 countries and created a highly balanced panel. The purpose of this paper is to examine the impact of the international health regulation index on state innovation capacity, and we used the GII index to measure state innovation capacity. The IHR core competence index was selected as the explanatory variable. Government effectiveness, political stability, and government corruption as indicators of negative government integrity were selected as moderators. Economic growth rate, trade situation, and urbanization rate were also included as control variables.

The variables and descriptions of this study are shown in Table 1. This paper draws the GII index from the World Innovation Capacity Development Report and the IHR index from the Global Health Organization^1^ Government corruption indexes, as well as governance effectiveness indexes, are derived from the World Bank^2^, while the IMF provides the National Economic Development Index and Export and Urbanization Rate Indicators^3^

**Table 1:** Data description.

**Type**	**Code**	**Description**
Outcome variable	GII	Global innovation index
Explanatory variable	IHR	WHO International Health Regulations (IHR) core competence index
Moderating variable	GE	Governance effectiveness index
	GS	Political stability index
	CORR	Government corruption index
Control variable	GDPG	Economic growth rate; GDP growth rate (%)
	EXP	Trade situation; exports/GDP (%)
	URBAN	Urbanization rate; urban population/total population (%)

Specifically, we refer to the WHO database when assessing the quality of public health policies. The WHO provides specific evaluations of healthcare system policies based on the degree to which different aspects of the IHR are implemented according to its wide range of data. A comprehensive legal framework is provided by the IHR that outlines the rights and obligations of states in dealing with health emergencies and public health events. Consequently, countries are required to report on their compliance with the IHR annually. Based on the level of development of public healthcare systems and compliance with the IHR framework, this dataset provides different scores assigned by WHO to a country. The WHO also sends a Surveillance Questionnaire every year to the IHR National Focal Points for data collection. Specifically, the questionnaire presents a list of 20 indicators designed to evaluate the various capacities of the public healthcare system. Data is then further processed to create an index indicating the percentage of attributes obtained. Based on compliance with IHR requirements, WHO assigns each country a score ranging from 0 to 100 for each category: 0 signifies no compliance, while 100 signifies full compliance with some IHR requirements.

The data from 145 countries were included in this research, and after the deletion of missing data points, a total of 739 sample points were obtained for 145 countries. Table 2 shows summary statistics of the main variables. Table 2 indicates that countries in our sample have differing levels of public health policy quality; likewise, state innovation capacity differs significantly across the countries we examined. The mean value of GII is 35.79, the minimum value is 2.35, the maximum value is 68.30, and the standard deviation is 13.61, indicating that the level of innovation index varies significantly among different countries. The mean value of the IHR index is 78.75, the minimum value is 0, the maximum value is 100, and the standard deviation is 31.37, indicating that the level of public health quality also varies significantly among different countries).

**Table 2:** Summary statistics.

**Variable**	***N***	**Mean**	**SD**	**Min**	**Max**
GII	739	35.97	13.61	2.350	68.30
IHR	739	78.75	31.37	0	100
GE	739	0.235	0.913	−1.566	2.241
GS	739	−0.0314	0.906	−2.677	1.616
CORR	739	0.112	1.010	−1.485	2.407
EXP	739	43.89	31.77	5.733	221.2
GDPG	739	3.650	2.833	−9.773	17.29
URBAN	739	61.53	22.22	11.19	100

### Model specification

We use a panel model instead of OLS cross-section regressions in our main analysis. Panel models have several advantages over cross-country regression models since the latter do not fully control for unobserved countries-specific effects. We estimated the model as follows to examine the relationship between IHR and state innovation capability:

$$\begin{array}{l} GII_{it}=\beta_{0}+\beta_{1}IHR_{it}+\beta_{i}X_{it}+\varepsilon_{it} \end{array}$$

Among them, *GII*_*it*_ represents the state innovation capacity, *IHR*_*it*_ represents the quality of public health policy (International Health Regulations Index), β_0_ is a constant term, ε_*it*_ is the random error term, *X*_*it*_ refers to the control variables of this paper. index *i* shows country i (values from 1 to 145, depending on the observed country) in time period t (values from 2010 to 2017). When β_1_ > 0, it means that the larger the international health regulation index, the stronger the state innovation capacity.

In addition, this paper examines the moderating effects of governance effectiveness, political stability, and government integrity. In order to test the moderating effect of governance effectiveness and innovation ability, this paper constructs the model as follows:

$$\begin{array}{l} GII_{it}=\beta_{0}+\beta_{1}IHR_{it}+\beta_{2}GE_{it}+\gamma IHR_{it}*GE_{it}+\beta_{i}X_{it}+\varepsilon_{it} \end{array}$$

Among them, *GII*_*it*_ represents the state innovation capacity, *IHR*_*it*_ represents the quality of public health policy, *GE*_*it*_ represents the governance effectiveness, β_0_ is a constant term, ε_*it*_ is the random error term, *X*_*it*_ refers to the control variables of this paper. When γ > 0, it means that the higher the governance effectiveness is, the more significant the IHR index is to the state innovation capacity.

In order to test the moderating effect of political stability and innovation ability, this paper constructs the following model:

$$\begin{array}{l} GII_{it}=\beta_{0}+\beta_{1}IHR_{it}+\beta_{2}GS_{it}+\gamma IHR_{it}*GS_{it}+\beta_{i}X_{it}+\varepsilon_{it} \end{array}$$

Among them, *GII*_*it*_ represents the state innovation capacity, *IHR*_*it*_ represents the quality of public health policy, *GS*_*it*_ represents the political stability, β_0_ is a constant term, ε_*it*_ is the random error term, *X*_*it*_ refers to the control variables of this paper. When γ > 0, it means that the higher the political stability is, the more significant the IHR index is to the state innovation capacity.

In order to test the moderating effect of corruption index and state innovation capacity, this paper constructs the following model:

$$\begin{array}{l} GII_{it}=\beta_{0}+\beta_{1}IHR_{it}+\beta_{2}CORR_{it}+\gamma IHR_{it}*CORR_{it}\\ +\beta_{i}X_{it}+\varepsilon_{it} \end{array}$$

Among them, *GII*_*it*_ represents the state innovation capacity, *CORR*_*it*_ represents government corruption (negative government integrity), β_0_ is a constant term, ε_*it*_ is the random error term, *X*_*it*_ refers to the control variables of this paper. When γ > 0, it means that the higher the government corruption is, the more significant the IHR index is to the state innovation capacity.

Pearson's correlation coefficients were estimated between independent variables to detect potential multicollinearity. Correlations were generally low to moderate, suggesting a low probability of multicollinearity. The results of these analyses are not presented here, but they can be provided on request. To obtain a measure of compliance with the International Health Regulations, we used the WHO aggregated data and several indicators.

## Results

### Correlation analysis

A Pearson correlation analysis was performed first to determine whether our variables of interest exhibit a linear relationship.

Table 3 indicates that IHR and GII are significantly positively correlated at the 1% significance level, indicating that public health policy quality and state innovation capacity have a positive relationship. The GE variable and GII were significantly positively correlated at the 1% significance level, indicating a strong positive relationship between government effectiveness and state innovation capacity. The GS variable and GII were also significantly positively correlated at the 1% significance level, indicating a positive relationship between political stability and state innovation capacity. The CORR variable and GII were significantly positively correlated at the 1% significance level, showing that there is a positive relationship between government integrity and innovation.

**Table 3:** Correlation matrix of key variables.

**Variable**	**GII**	**IHR**	**GE**	**GS**	**CORR**	**EXP**	**GDPG**	**URBAN**
GII	1							
IHR	0.383^***^	1						
GE	0.824^***^	0.323^***^	1					
GS	0.616^***^	0.171^***^	0.752^***^	1				
CORR	0.777^***^	0.261^***^	0.942^***^	0.769^***^	1			
EXP	0.418^***^	0.130^***^	0.464^***^	0.483^***^	0.416^***^	1		
GDPG	−0.303^***^	−0.089^**^	−0.312^***^	0.203^***^	−0.295^***^	−0.038	1	
URBAN	0.595^***^	0.340^***^	0.640^***^	0.481^***^	0.600^***^	0.367^***^	−0.326^***^	1

t-statistics in parentheses.

*** p < 0.01.

### Regression analysis

Following the correlation analysis, we conducted a fixed effects regression to further examine the impacts of public health policy quality on state innovation capacity.

Table 4 shows our estimation results using the fixed effect model. We start by estimating the relationship between public health policy quality and state innovation capacity. We added government effectiveness, political stability, government integrity, and control variables in the subsequent models. The positive coefficient of IHR suggests that higher quality of public health policy is related to a higher level of state innovation capacity, and the relationship remains robust and significant after introducing other variables. Specifically, a unit increase in the quality of public health policy results in a 0.555 unit increase in the state innovation capacity. Furthermore, the results indicate that there is no direct relationship between government effectiveness or government integrity and state innovation capacity; however, they may have indirect effects that should be explored. In terms of the controls, trade and urbanization are significantly related to state innovation capability, suggesting that a more prevalent trade system and a higher level of urbanization are associated with greater innovation capability.

**Table 4:** Effects of public health policy quality on state innovation capacity.

Variable	−1
	mm1
	GII
IHR	0.555^***^
	−4.623
GE	1.119
	−0.441
GS	2.538^*^
	−1.891
CORR	0.791
	−0.351
EXP	0.232^***^
	−4.82
GDPG	−0.177
	(−1.528)
URBAN	2.063^***^
	−8.226
Constant	−105.111^***^
	(−6.700)
Observations	739
Number of id	145
R-squared	0.184

t-statistics in parentheses.

*** *p* < 0.01,

* *p* < 0.1.

### Moderation analysis

To investigate the impacts of government effectiveness, political stability, and government integrity, we performed a moderation test on the model.

Table 5 shows the estimation results. Our findings suggest that there is no conditional effect of government effectiveness (column 1). The positive and significant interaction effect (column 2) suggests that in countries with higher political stability, the quality of public health policies has a more significant positive effect on state innovation capacity. In contrast, as indicated by the interaction term IHR_CORR in column 3, public health policies have a more significant negative effect on state innovation capacity in countries with higher levels of government corruption. Therefore, in a country with cleaner governance, the quality of public health policy has a greater positive impact on the development of state innovation capacity.

**Table 5:** Interaction effects between public health policy quality and government effectiveness, political stability, and government integrity.

**Variable**	**Model 1**	**Model 2**	**Model 3**
	**GII**	**GII**	**GII**
IHR_GE	−0.015		
	(−0.957)		
GE	2.489	1.586	1.440
	(0.855)	(0.625)	(0.569)
IHR_GS		0.026^*^	
		(−1.899)	
GS	2.571^*^	4.271^***^	2.534^*^
	(1.914)	(2.635)	(1.893)
IHR_CORR			−0.028^**^
			(−2.091)
CORR	0.548	0.505	2.587
	(0.241)	(0.224)	(1.074)
IHR	0.553^***^	0.548^***^	0.648^***^
	(4.311)	(3.860)	(3.815)
EXP	0.242^***^	0.247^***^	0.251^***^
	(4.912)	(5.073)	(5.142)
GDPG	−0.178	−0.193^*^	−0.179
	(−1.536)	(−1.658)	(−1.550)
URBAN	2.064^***^	2.074^***^	2.075^***^
	(8.230)	(8.286)	(8.296)
Constant	−105.325^***^	−105.800^***^	−105.893^***^
	(−6.713)	(−6.757)	(−6.768)
Observations	739	739	739
R-squared	0.186	0.189	0.190
Number of country	145	145	145

t-statistics in parentheses.

*** *p* < 0.01,

** *p* < 0.05,

* *p* < 0.1.

### Robustness analysis

We performed a robustness test in this section. The GDP growth rate in the control variable is replaced by the per capita GDP growth rate (PGDPG), and the re-regression results of the model in this paper are shown in Table 5. Table 6 shows that our main results are robust for using this alternative measurement.

**Table 6:** Effect of public health policy quality on state innovation capacity with an alternative measurement.

**Variable**	**(1)**
	**mm1**
	**GII**
IHR	0.555^***^
	(4.556)
GE	1.191
	(0.471)
GS	2.456^*^
	(1.833)
CORR	0.845
	(0.375)
EXP	0.232^***^
	(4.849)
PGDPG	−0.204^*^
	(−1.734)
URBAN	2.066^***^
	(8.246)
Constant	−105.448^***^
	(−6.730)
Observations	739
Number of id	145
R-squared	0.185

t-statistics in parentheses.

*** *p* < 0.01,

* *p* < 0.1.

## Regression discontinuity design and endogeneity tackling

### The selection of running variable and the search of discontinuity

In the part of endogeneity tacking, we adopted the Regression Discontinuity Design (RDD), which can create an opportunity for an “quasi-experimental survey” within controlled samples around the cut-off point (90). The underlying thought of RD designs originates from the Neyman-Rubin model trying to infer the effect of treatment by comparing those really-treated individuals with “counterfactual themselves” (91).

However, in the social sciences, we can't observe an individual both under the treatment Yi1 and the control Yi0 at the same time. And in our panel data, a country's capability indeces, such as governance effectiveness, political stability and government corruption, would be regarded as fixed variables during a controlled period. That means it is unrealistic to directly observe the cause-effect (Yi1-Yi0) of a state level unit. But the approach of RDD can mitigate these methodological dilemmas. Since 1990, RD designs have been a common casual effect estimation in economics (92–94). And in political science, it has been a practical approach to approximate the effect of public policies. Mostly, the running variables of these studies are related to population (95). And in political science, RDD usually has been used to analyze the effect of public policies and elections analyses (96). Generally, there is a boundary between the control group and treatment group whose score is divided by the cutoff. And if some units belong to treatment group but its score is below the cutoff and/or conversely, the RDD should be fuzzy instead of the clear (97).

In our dataset, the units are existing countries, which are considered as unrandomized observations in principal. Due to its potential endogeneity, we can't exactly find out the clear boundary between the countries with higher governance effectiveness and those with lower, neither the precise comparisons in terms of the indexes like political stability and political corruption. Therefore, in our study, we decided to take the fuzzy RDD according to the nature of variables.

In detail, we collected the Fragile State Index (FSI) from The Fund for Peace as our running variable. FRI could be seen as a reliable overall matching score calculated by 12 indicators that encompass estimation from social respect to economic, political and military respect on 198 countries' fragility situation (98). Every indicator is scaled from 0 (worst) to 10.0 (best), and the total score varies from 0 (least fragile) to 12.0 (most fragile). And it is worth mentioning that the indicator 7 (state legitimacy), the 8 (public services), the 9 (human rights and rule of law) and the 11 (factionalized elites) can reflect simultaneously the situation of one country's governance effectiveness, political stability and government corruption to a certain extent. Hence, in the present study, we took the 8 year's average score of FSI (from 2010 to 2017) as the running variable for our design.

And we have 3 reasons for choosing such dataset:

Firstly, the time span of the dependent variable is from 2010 to 2017, hence the FSI should also include this period. And in the present study, we matched every unit with the corresponding FSI by year. However, due to the lack of developed countries, the possibility of sampling error cannot be ruled out in the 2010 data, so we excluded the 2010 data in the coming RDD.

**Figure 1:**
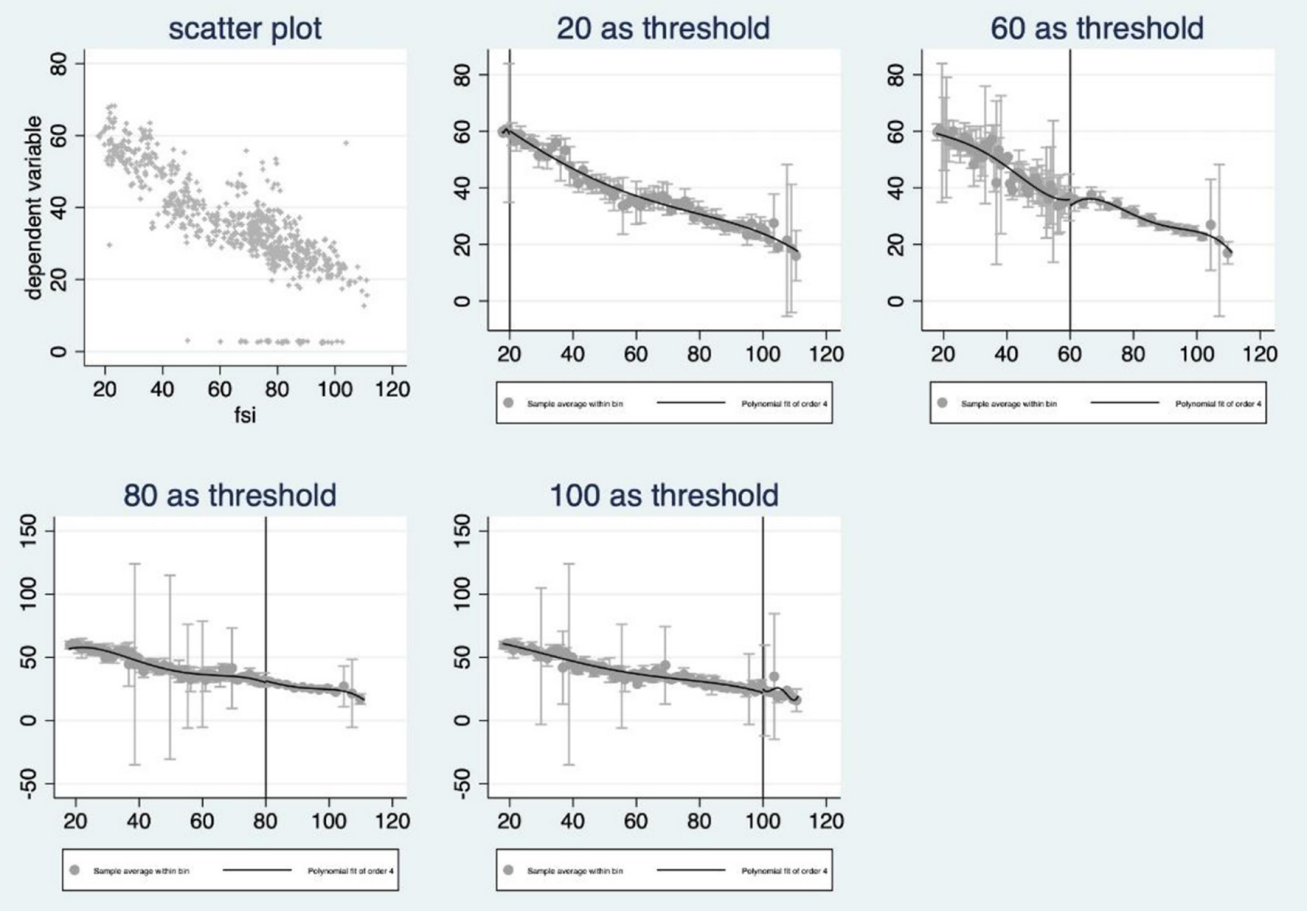
General scatter and fitting results with different thresholds.

Secondly, the cutoff of this running variable is given, and according to the index scale, countries with FSI higher than 60 would be seen as warming-level fragile countries or worse, compared with those relatively stable or even better countries with FSI below 60. And we can't deny the possibility of bias caused by subjective assume as taking the so-called 60 as the threshold, therefore, the fuzzy RDD was took into consideration in this study. And when we took a try after setting the 60 as the cutoff, the discontinuity is not significant because the two closest confidence intervals to the left and right of the threshold overlap, and so are other given cutoff points (20, 60, 80, 100, see Figure 1).

However, once set 40 as the cutoff, the discontinuity would be significant (see chart below). According to the official definition from The Fund for Peace, countries belong to the band 40–60 are “stable countries” where those below 40 are countries more stable but still not sustainable while those above 40 are still stable but recently may suffer from some social problems.

More precisely, the countries below 40 are generally those best developed countries like Japan and Singapore, while those below 60 normally are still developed countries and least developed countries but with emerging social problems or quasi developed countries like Spain, Poland and Czech Republic. This accords with our empirical cognition.

Finally, those countries, around the cutoff but within the set band width, are normally similar in population, territorial size, economic growth rate, trade situation and urbanization rate. In this setting, we picked out those countries as similar as possible but divided into the stable group (treatment group) and fragile group (control group) merely due to their differences in the FSI, then we could estimate the causal effect of the public health policies quality on the state innovation capability, though the external validity is limited.

After collecting this dataset, we standardized the average FSI of each unit (average FSI minus 60) to let 0 become the “net” cutoff, and then left all of them multiplied by−1, thus we can see a standardized distribution with fragile group on the left of cutoff and stable group on the right. Next, we used STATA as our instrument to proceed the design.

**Figure 2:**
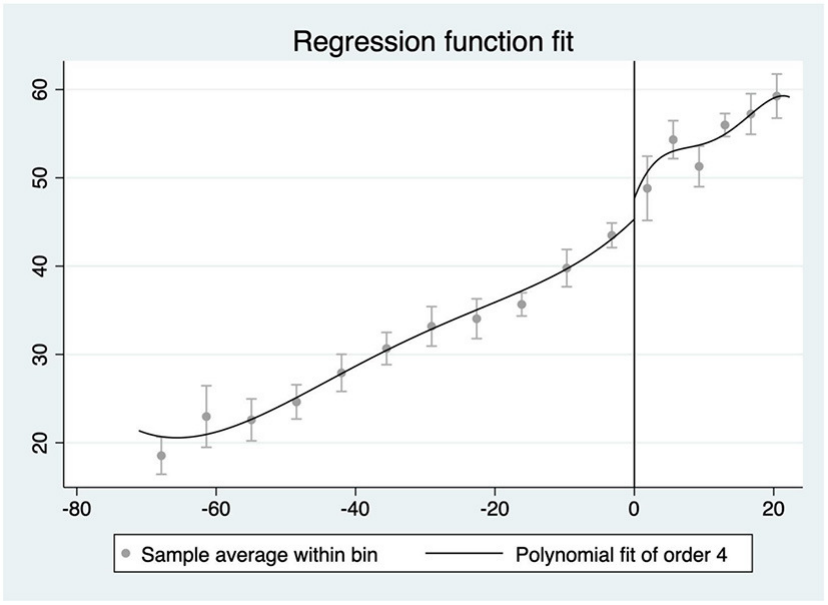
Polynomial fitting (40 as threshold).

In the first place, we set a 95% confidence interval to find if there is a clear discontinuity at the cutoff. Here, we have tried polynomial fit of order 4 and the result is significant that around the threshold (FSI = 40) there is a discontinuity making the state innovation capacity changed abruptly (see Figure 2).

### Local linear regression

We assumed that the treatment effect in the breakpoint neighborhood would be linear, and thus we identified it by performing linear regression on the left and right parts respectively and comparing the difference of regression coefficients between the two sides. An important part of local regression test is the selection of the size of the breakpoint neighborhood, which is also known as the trade-off problem of bandwidth selection in RDD analysis. This is because the larger the bandwidth, the more samples will be included in the test, and the parameter estimation will be more accurate, but it also means that the requirement of sample randomness is more difficult to meet, and the endogeneity problem may be more serious.

The neighborhood of the breakpoint Xc in this paper is ([XC-H0, Xc + H1]), and H0 and H1 are left and right bandwidths, respectively. H0 and H1 could be the same, or they could be different. And STATA's breakpoint analysis instructions automatically give the optimal bandwidth. And we can see that the coefficient of the treatment effect is 5.1715 where the bin width was set as 7.099 above/below the cutoff (see chart below), which means once other variables controlled, around the point 40 of FSI, those countries with relatively more stable domestic situation but not belonging to those best, on average, are 5.1715 higher on the GII, than those countries with relative less stable domestic situation (see Table 7).

**Table 7:** RD analysis-linear fitting.

**Method**	**Coef**.	**Std. Err**.	***z***	***P > z***	**95%Conf**.	**Interval**
Conventional	5.1715	2.1944	2.3567	0.018	0.870608	9.47239
Robust	-	-	1.9719	0.049	0.031788	10.5117

### Local polynomial regression

Considering that the linear assumption may misestimate the regression coefficients around the breakpoint, we adopted the method of nonlinear fitting to compensate, that is, using the local polynomial breakpoint regression method. And when the quadratic fitting was used, the result is still significant though with a higher coefficient (6.6373, see chart below), which means the existing of breakpoint is clear but in each group there would be some “extreme” units that may to some extent influence but not disturb the robustness of difference caused by the effect of state frangibility, which means once other variables controlled, around the point 40 of FSI, those countries with relatively more stable domestic situation but not belonging to thoese best, on average, are 6.6373 higher on the GII, than those countries with relative less stable domestic situation. It is worth noting that the coefficient calculated by polynomial regression is 1.4658 higher than the linear result though both of them are significant. However, it's not strange because the observation unit is country rather than individuals, that means we may not theoretically exclude the influence caused by “extreme units” due to limited amount of observation (see Table 8).

**Table 8:** RD analysis-polynomial fitting.

**Method**	**Coef**.	**Std. Err**.	***z***	***P > z***	**95%Conf**.	**Interval**
Conventional	6.6373	3.0784	2.156	0.031	0.603639	12.6709
Robust	-	-	2.025	0.043	0.234827	14.3995

### Test of design's validity

**Figure 3:**
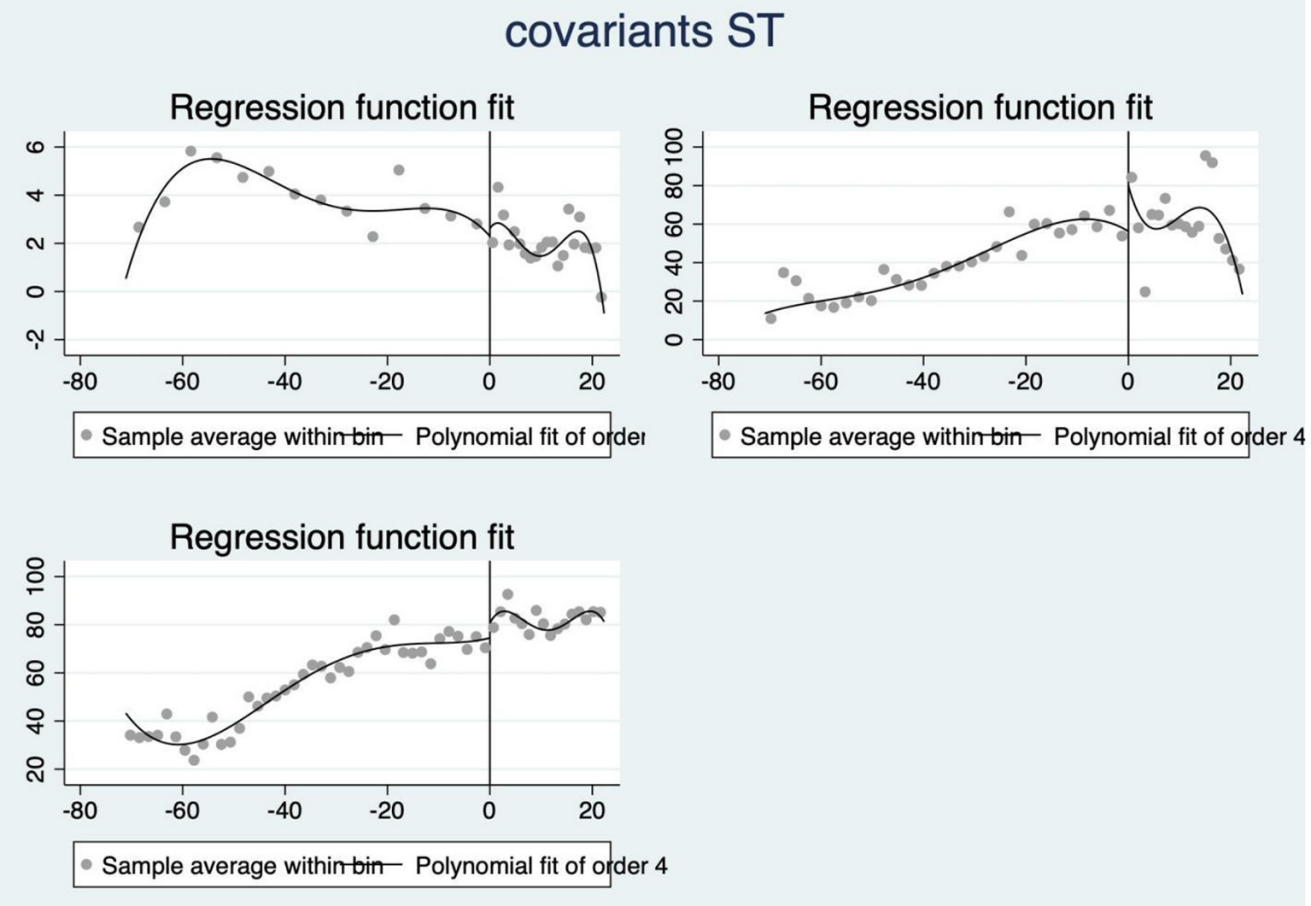
Smoothness test of covariant.

Firstly, a test of smoothness is necessary. Samples near the threshold will be considered as similar as possible if there are no sharp jumps in all attributes (except the outcome variable). Therefore, we used the assumption of local smoothing, with each covariate included in the RD test as a placebo outcome variable. And in this study, there are three covariants: economic growth rate (GDPG), exports-GDP rate (EXP) and urbanization rate (URBAN). And the visualized result of three covariants' smoothness test by order 4 lies below (see Figure 3).

Though each of three results seems broken by the threshold, we still can't say it is a strict result to deny the smoothness. Therefore, we respectively took 3 sets of regression analysis where we put the covariant as the outcome variable while still using the standardized FSI as running variable. And the results are not significant (with *P*-value respectively being 0.311, 0.188 and 0.218), which means we can not reject the assume that the discontinuity doesn't exist (see charts below). On this basis, we could declare that the covariants of the observations are not abrupt (see Tables 9–11).

**Table 9:** Placebo test (GDPG as outcome variable).

**Method**	**Coef**.	**Std. Err**.	***z***	***P* > *z***	**95%Conf**.	**Interval**
Conventional	−1.3057	1.2878	−1.0139	0.311	−3.82987	1.21838
Robust	-	-	−1.173	0.241	−4.58331	1.15127

**Table 10:** Placebo test (EXP as outcome variable).

**Method**	**Coef**.	**Std. Err**.	***z***	***P* > *z***	**95%Conf**.	**Interval**
Conventional	24.821	18.857	1.3163	0.188	−12.1375	61.7786
Robust	-	-	1.4517	0.147	−10.9645	73.5928

**Table 11:** Placebo test (URBAN as outcome variable).

**Method**	**Coef**.	**Std. Err**.	***z***	***P* > *z***	**95%Conf**.	**Interval**
Conventional	8.2717	6.7161	1.2316	0.218	−4.89169	21.435
Robust	-	-	0.8099	0.418	−8.75164	21.0785

## Discussion

Overall, the empirical results of this study demonstrate a positive and statistically significant relationship between the quality of public health policy as an independent variable and the state innovation capacity as a dependent variable. The quality of public health policies has a substantial and significant impact on state innovation capacity. These findings confirm the main hypothesis of this study. We believe that the policy toolkit plays a significant role in the relationship between policy and innovation, with public health policy not only forming part of the field of public health but also in the integration of policy tools into many different areas. When devising and implementing public health policies, efficient public health policies produce more stable long-term innovation strategies and can better invest and implement measures to ensure the success of strategies. The purpose of public health policies is to ensure a high degree of coherence of new inventions, new technologies, and new products developed by enterprises; when public health investment has increased significantly, public health policies reduce general rent-seeking risks and improve innovation performance.

Nevertheless, we discovered some interesting findings when discussing the role of moderating variables in our model. In other words, the three moderating variables of governance effectiveness, political stability, and government integrity produced different statistically significant results. The results of this model show that political stability and government integrity are moderating variables that can explain the causal relationship between independent and dependent variables. These results complement the literature discussion on the determinants of public policy quality and state innovation capacity from the perspective of policy toolkits and consider adding additional moderating variables to expand the model.

The results showed that when governance effectiveness varies, there is little difference in the impact of public health policy quality on state innovation capacity, so Hypothesis 2 could not be supported. There may be an explanation why governance effectiveness does not have a significant impact on this relationship in that, whether the PPP mechanism, the market resource allocation mechanism, or the government regulatory mechanism, they are not directly related to the state innovation capacity, especially the PPP mechanism and the government regulatory mechanism. Consequently, there is no incentive to enhance state innovation capacity. This finding challenges the previous view of innovation literature (46).

Secondly, in countries with greater political stability, the quality of public health policies is more likely to have a positive influence on state innovation capacity, so our Hypothesis 3 hold true. The quality of the policy of the state is one aspect that ensures the production of public goods that satisfy the public in terms of both quantity and quality, and one of the factors for ensuring the production of public goods is the quality of public policies. The quality of public health policy is of increasing importance in the contemporary world where public health crises are commonplace. It is imperative to have a stable political environment in order to foster the development of science and technology inside a country as well as international exchange of science and technology. This study contributes to the literature on the relationship between political stability and state innovation capacity (48).

Thirdly, countries with a higher degree of government integrity are more likely to have a positive impact on the development of state innovation capacity due to the quality of public health policies. It is possible that the negative correlation between public policy quality and government rent-seeking is due to the fact that the process of formulating and implementing public policies reinforced by supervision has successfully curbed the phenomenon of government rent-seeking. Further, we believe that when the cost of government rent-seeking increases significantly, manufacturers must convert the price of bribery into innovation costs, which will increase the added value of products in order to respond to market competition. There has been an absence of attention to the role of government integrity in the relationship between policy quality and national innovation capacity in public health (49, 52). This study identified the moderating role of government integrity in this relationship, and the findings of this research are likely to provide theoretical support for contemporary state process design and process decision making.

Finally, as the boundaries between the indicators discussed above (e.g., corruption and integrity, stability and instability, efficiency and inefficiency) are not clear, there may be a greater degree of endogeneity. It is therefore difficult to directly assert that the implementation of high-quality public health policies in clean (stable or efficient) countries will necessarily lead to innovation. To address the above endogeneity issues, we added a breakpoint regression approach. It is found that the above mechanism may only be relevant between countries that are more politically stable and those that are most politically stable. Our model is based on the empirical analyses of a number of factors that are strongly related to state innovation capacity, including economic growth rate, urbanization, and national trade (99–101). Moreover, we tested robustness using the per capita GDP growth rate instead of GDP growth (101), and we found that the positive impact of public health policy quality on state innovation capacity remains significant (102). We were surprised to find that there was no apparent difference in the impact of the quality of public health policies on the ability of countries to innovate when there were variations in governance effectiveness. This study concludes that there is insufficient evidence to dismiss the positive impact of public health policy quality on a nation's ability to innovate^4^.

## Conclusion

Using a sample of 145 countries observed between 2010 and 2017, this study examines the impact of public health policy quality (IHR) on state innovation capacity (GII). After controlling for other variables, we observed statistically significant evidence that public health policy quality (IHR) influences state innovation capacity (GII) under the influence of moderating factors.

The results of this study have some practical implications as well. Firstly, it fills research gaps in the relationship between public health sector policy formulation and implementation and country-specific innovation-potentially informative for policymakers and designers of policies and programs that governments, particularly from the health sector and the science and technology sectors, can use the findings of this study to enhance the innovation factor in public health policy formulation and implementation. We also use governance effectiveness, political stability, and government integrity as moderating variables, and conclude that political stability and government integrity play a key moderating role in this relationship. The findings may also provide guidance for government policymaking. Furthermore, it provides an empirical quantitative analysis of policy quality in the public health sector, which is lacking in most cases. This paper, on the other hand, quantitatively measures the quality of national public health policies by utilizing WHO's IHR data. It is the third contribution of this paper that shows that state innovation capacity is enhanced not only by economic growth, urbanization, R&D investment, and foreign trade but also by public policy, especially public health policy. Many pieces of literature have discussed the positive effects of policy quality on state capacity (S&T), but these tend to select policies closely related to the S&T domain (106, 107), ignoring health policy as an infrastructural role of policy on state innovation capacity (108), which is increasingly important during COVID-19 era. Therefore, our study fills this gap.

Furthermore, we found in RD that the mechanisms discussed above may only make sense between the more politically stable countries and the most politically stable countries. This finding challenges the existing mainstream political economy community's research on the causal relationship between policy quality and state capacity. Current scholars generally agree that in authoritarian (mostly developing) countries, COVID-19 improvements in health policy quality strengthen state capacity (109, 110). However, in developing countries with relative political instability, our empirical evidence suggests that the impact of health policy quality on state innovation capacity is insignificant, just as some scholars find that health corruption in developing countries in the context of epidemics is likely to be a significant cause of cannibalization of state innovation capacity (111). The issue will probably be an important direction for this study in the future.

## Data availability statement

Publicly available datasets were analyzed in this study. This data can be found here: The Gloal health Organization IHR report https://www.who.int/data/gho/data/indicators/indicator-details/GHO/legislation, The World Bank http://info.worldbank.org, and IMF https://www.imf.org.

## Author contributions

XJ contributed to the execution of the experimental research. LG and ZL participated in completing the data analysis and writing the manuscript. XJ, LG, and ZL contributed in experimental design. SH and CC participated in the analysis of the experimental results. LL participated in the project design, was the person in charge, and guided experimental design. BZ and JC participated in data analysis, thesis writing, and revision. All authors have read and agreed to the final text.

## Funding

This research was funded by the Ministry of Education China, grant number 21YJC790033.

## Conflict of interest

The authors declare that the research was conducted in the absence of any commercial or financial relationships that could be construed as a potential conflict of interest.

## Publisher's note

All claims expressed in this article are solely those of the authors and do not necessarily represent those of their affiliated organizations, or those of the publisher, the editors and the reviewers. Any product that may be evaluated in this article, or claim that may be made by its manufacturer, is not guaranteed or endorsed by the publisher.

## Footnotes

^1^The Global Health Organization IHR report https://www.who.int/data/gho/data/indicators/indicator-details/GHO/legislation.

^2^The World Bank http://info.worldbank.org.

^3^IMF https://www.imf.org.

^4^Additionally, the specific circumstances demonstrating the independence of the GII from several external countries reinforce our recommendation that policymakers play a major role in supporting the development of the IHR. Policy recommendations from other studies can be drawn from this; it is particularly important to improve the quality of policies in the public health sector, where policymakers should focus on forming and implementing policies that support the diffusion of health care services throughout society (103, 104). Therefore, the state innovation capacity affects policy design and implementation, and vice versa (2, 105).
